# Supplementary material for: A decade of theory as reflected in Psychological Science (2009–2019)
Source: PLoS One. 2021 Mar 5;16(3):e0247986. doi: 10.1371/journal.pone.0247986 (PMC7935264; doi:10.1371/journal.pone.0247986)
Supplement: S1 File — (DOCX) [file pone.0247986.s001.docx]

**A decade of theory as reflected in *Psychological Science* (2009-2019)**

**Contents**

**Page Description**

1 Coding criteria for the identification of psychological theories

2 S1 Table. Percentage of Psychological Science articles from 2009-2019 using specific words according to year.

S2 Table. Complete counts of the use of theory, theories, preregistration, and naming a theory for 11 years of published articles.

3 S3 Table. Complete counts of studies that name a specific theory against preregistered status.

S4 Table. Percentage of studies that named a specific theory according to whether that study was preregistered or not organized by year.

S5 Table. Contingency table of counts of studies that name a specific theory against the main type of data recorded.

S6 Table. Percentage of studies that named a specific theory according to the primary type of data recorded in that study.

4 S7 Table. Names and frequency of use for theories mentioned in *Psychological Science* from 2009-2019.

13 S8 Table. Total number of studies using the word hypothesis correctly and incorrectly according to year.

**Coding procedure and criteria for the identification of psychological theories.**

All 2,225 articles were examined individually. Each PDF was opened and a word search was conducted for the string “theor”. The context around each instance of that string was examined, along with the abstract and the keywords.

Because the goal was to expand this part of the investigation to include all of the theories that people were talking about, there was slightly different criteria than that used for the other coding tasks in this study. The criteria were as follows:

1. Any time a theory was named or mentioned directly in-text it was counted.

2. This also included terms labelled “model” or “hypothesis” as long as a specific explanation or prediction was discussed and if it was clear that *theory* was being used to refer to the mentioned model or hypothesis or that they were treating it as a theory.

3. This also included instances where the theory was referred to indirectly. For example, “Attachment theorists have long suggested that…” or was referred to only by the name or a single citation (e.g., “According to Bandura…”) only as long as a specific explanation or prediction was discussed. Additionally, it was counted as long as it referred specifically to the theory (e.g., “Game-theorists…” or “attachment theorists”). That is, “relationship theorists…” or “personality-theoretical accounts…” would not be counted because there is no *relationship theory* or *personality theory.* In line with this, “Game-theoretical” or “game-theorizing” was always counted, as it was used to refer to a specific effect, prediction, or decision derived only from game theory.

4. We did not count instances where the word (theory or theorized) was used in place of “hypothesized” (eg., “Our theory was that…” or “Stevens theorized that…”) or instances only in the references. This was often encountered when authors would refer to “Our theory” but had not named or verbally formalised the theory. Instances like “Another theory suggests…” followed by more than one in-text citation would not be counted.

5. Evolutionary theory was counted when it included an in-text citation to Darwin or mentioned a specific prediction or sub-model (e.g., sexual selection). The term “evolutionary theory” on its own (e.g., “These experiments can inform evolutionary theory.”) was not counted.

6. Topics of research were not counted. For example, “Theory of mind” was not counted, nor was “conspiracy theory”, “folk theory” or “lay theory” because these are topics of study rather than scientific theories that can be used to make a specific prediction. The same was true for “learned helplessness” which was referred to at least once as a theory but actually refers to a specific effect, so it was not counted. Additionally, “theories of embodiment” (along with “embodied cognition” and “theories of embodiment”) was not counted because this was never mentioned as a singular theoretical construct, but rather as a *topic* of research. Mathematical theorems (e.g., Bayes theorem, central limit theorem) were also not counted.

7. Finally, sometimes authors gave classes of theories or observed effects their own names for the purposes of rhetoric—for example, “We call this the *inclusion theory…*” Most often this referred to an effect that was common across multiple different named theories, and was not counted. Sometimes it was used to generally refer to a characteristics of theories (e.g., “Dynamic theories of personality development”) and this was also not counted.

**S1 Table. Percentage of Psychological Science articles from 2009-2019 using specific words according to year.**

| Year | Total | Preregistered | Theory | Theories | Theoretical | Named a Theory | Tested a Theory |
| --- | --- | --- | --- | --- | --- | --- | --- |
| 2009 | 192 | 0.00 | 53.65 | 32.81 | 32.81 | 30.73 | 21.88 |
| 2010 | 284 | 0.00 | 64.08 | 22.89 | 20.07 | 20.07 | 6.34 |
| 2011 | 232 | 0.00 | 39.66 | 28.45 | 27.59 | 21.12 | 6.90 |
| 2012 | 213 | 0.00 | 64.79 | 30.52 | 26.76 | 28.64 | 14.55 |
| 2013 | 304 | 0.00 | 40.46 | 32.89 | 37.83 | 20.39 | 13.82 |
| 2014 | 242 | 0.00 | 44.63 | 27.27 | 31.40 | 23.14 | 8.26 |
| 2015 | 178 | 0.56 | 41.01 | 29.21 | 37.08 | 19.66 | 6.18 |
| 2016 | 144 | 2.78 | 75.00 | 36.11 | 32.64 | 33.33 | 17.36 |
| 2017 | 145 | 8.28 | 66.21 | 27.59 | 40.00 | 26.21 | 9.66 |
| 2018 | 156 | 20.51 | 74.36 | 34.62 | 44.87 | 26.28 | 9.62 |
| 2019 | 135 | 25.19 | 40.74 | 29.63 | 41.48 | 24.44 | 8.89 |

**S2 Table. Complete counts of the use of theory, theories, preregistration, and naming a theory for 11 years of published articles.**

| Year | Theory | Named a Theory | Theories | Preregistered |
| --- | --- | --- | --- | --- |
| 2009 | 103 | 59 | 63 | 0 |
| 2010 | 182 | 57 | 65 | 0 |
| 2011 | 92 | 49 | 66 | 0 |
| 2012 | 138 | 61 | 65 | 0 |
| 2013 | 123 | 62 | 100 | 0 |
| 2014 | 108 | 56 | 66 | 0 |
| 2015 | 73 | 35 | 52 | 1 |
| 2016 | 108 | 48 | 52 | 4 |
| 2017 | 96 | 38 | 40 | 12 |
| 2018 | 116 | 41 | 54 | 32 |
| 2019 | 55 | 33 | 40 | 34 |

**S3 Table. Contingency table of counts of studies that name a specific theory against preregistered status.**

| Pre-registered | No specific theory | Specific theory | Did not test theory | Tested theory |
| --- | --- | --- | --- | --- |
| Not pre-registered | 502 | 152 | 570 | 84 |
| Pre-registered | 64 | 19 | 70 | 13 |

**S4 Table. Percentage of studies that named a specific theory according to whether that study was preregistered or not organized by year.**

| Year | Theory named? | Not Pre-registered | Pre-registered |
| --- | --- | --- | --- |
| 2014 | No specific theory | 63.64 | 0 |
|  | Specific theory | 36.36 | 0 |
| 2015 | No specific theory | 69.74 | 0 |
|  | Specific theory | 30.26 | 0 |
| 2016 | No specific theory | 64.00 | 66.67 |
|  | Specific theory | 36.00 | 33.33 |
| 2017 | No specific theory | 58.33 | 100.00 |
|  | Specific theory | 41.67 | 0.00 |
| 2018 | No specific theory | 62.07 | 75.00 |
|  | Specific theory | 37.93 | 25.00 |
| 2019 | No specific theory | 58.33 | 50.00 |
|  | Specific theory | 41.67 | 50.00 |

**S5 Table. Contingency table of counts of studies that name a specific theory against the main type of data recorded.**

| Specific theory | Self-report or Survey | Biological or Physiological | Observational or Behavioural | Other |
| --- | --- | --- | --- | --- |
| No specific theory | 655 | 142 | 354 | 63 |
| Specific theory | 232 | 35 | 101 | 22 |

**S6 Table. Percentage of studies that named a specific theory according to the primary type of data recorded in that study.**

| Year | Theory named? | Self-report/Survey | Bio/Physio | Observational | Other |
| --- | --- | --- | --- | --- | --- |
| 2009 | No specific theory | 55.88 | 66.67 | 52.17 | 50.00 |
|  | Specific theory | 44.12 | 33.33 | 47.83 | 50.00 |
| 2010 | No specific theory | 59.68 | 84.62 | 76.47 | 50.00 |
|  | Specific theory | 40.32 | 15.38 | 23.53 | 50.00 |
| 2011 | No specific theory | 64.06 | 66.67 | 54.84 | 0.00 |
|  | Specific theory | 35.94 | 33.33 | 45.16 | 100.00 |
| 2012 | No specific theory | 53.25 | 14.29 | 35.71 | 0.00 |
|  | Specific theory | 46.75 | 85.71 | 64.29 | 100.00 |
| 2013 | No specific theory | 67.69 | 58.82 | 68.18 | 77.78 |
|  | Specific theory | 32.31 | 41.18 | 31.82 | 22.22 |
| 2014 | No specific theory | 56.72 | 84.62 | 75.00 | 50.00 |
|  | Specific theory | 43.28 | 15.38 | 25.00 | 50.00 |
| 2015 | No specific theory | 64.29 | 87.50 | 70.97 | 66.67 |
|  | Specific theory | 35.71 | 12.50 | 29.03 | 33.33 |
| 2016 | No specific theory | 58.82 | 81.25 | 55.56 | 70.00 |
|  | Specific theory | 41.18 | 18.75 | 44.44 | 30.00 |
| 2017 | No specific theory | 67.74 | 60.00 | 60.00 | 50.00 |
|  | Specific theory | 32.26 | 40.00 | 40.00 | 50.00 |
| 2018 | No specific theory | 61.70 | 62.50 | 78.95 | 62.50 |
|  | Specific theory | 38.30 | 37.50 | 21.05 | 37.50 |
| 2019 | No specific theory | 52.94 | 57.14 | 57.14 | 60.00 |
|  | Specific theory | 47.06 | 42.86 | 42.86 | 40.00 |

**S7 Table. Names and frequency of use for theories mentioned in *Psychological Science* from 2009-2019.**

| **Name** | **2009** | **2010** | **2011** | **2012** | **2013** | **2014** | **2015** | **2016** | **2017** | **2018** | **2019** | **Count** |
| --- | --- | --- | --- | --- | --- | --- | --- | --- | --- | --- | --- | --- |
| Achievement Goal Theory | 1 |  |  |  |  |  |  |  |  |  |  | 1 |
| Achievement Motivation |  | 1 |  |  |  |  |  |  |  |  |  | 1 |
| ACT-R Declarative Memory Model |  |  |  |  |  | 1 |  |  |  |  |  | 1 |
| Action Control Theory |  |  |  |  |  |  |  |  |  | 1 |  | 1 |
| Action Identification |  |  |  | 1 |  |  |  |  |  |  |  | 1 |
| Adaptive Calibration Model |  |  |  |  |  | 1 |  |  |  |  |  | 1 |
| Adaptive Gain Theory |  |  |  |  |  |  |  |  |  |  | 1 | 1 |
| Affect As Information Theory |  |  | 1 |  |  | 1 |  |  |  |  |  | 2 |
| Affect Valuation Theory |  |  |  |  |  |  |  |  |  | 1 |  | 1 |
| Agency Theory | 1 |  | 1 |  |  |  |  |  |  |  |  | 2 |
| Alcohol Myopia Theory | 1 |  |  |  |  |  |  |  |  |  |  | 1 |
| Allostasis Theory |  |  |  | 1 | 1 |  |  |  |  |  | 1 | 3 |
| Ambivalent Sexism Theory |  |  |  |  |  |  |  |  |  |  | 1 | 1 |
| Anchoring Theory |  |  | 1 |  |  |  |  |  |  |  |  | 1 |
| Appraisal Theory |  |  | 1 |  |  |  | 1 |  |  | 1 |  | 3 |
| Approach/Inhibition Theory Of Power | 1 | 1 |  |  |  |  |  | 1 |  |  |  | 3 |
| Approximate Number Sense |  |  |  |  |  | 1 |  |  |  |  |  | 1 |
| Arousal Inhibition Theory |  |  |  |  |  |  |  |  |  |  | 1 | 1 |
| Arousal-Biased Competition |  |  |  | 1 |  | 2 |  |  |  |  |  | 3 |
| Associative Accumulation Model |  |  |  |  |  |  |  |  |  | 1 |  | 1 |
| Associative Learning Theory |  | 1 |  |  |  |  |  |  |  | 1 |  | 2 |
| Attachment Theory | 1 | 5 |  | 2 | 2 |  |  | 3 | 2 | 2 |  | 17 |
| Attention Theory |  |  | 1 |  |  |  |  |  |  | 1 |  | 2 |
| Attentional Control (Anxiety) |  |  |  |  |  |  |  |  |  | 1 |  | 1 |
| Attentional Control (VWM) |  |  |  | 1 |  |  |  |  |  |  |  | 1 |
| Attentional Engagement Theory |  |  |  |  | 1 |  |  |  |  |  |  | 1 |
| Attentional-Gate Theory Of Timing | 1 |  |  |  |  |  |  |  |  |  |  | 1 |
| Attribution Of Competence (Anchoring) |  |  |  |  |  |  |  | 1 |  |  |  | 1 |
| Attribution Theory | 1 |  |  |  |  |  |  | 1 |  | 1 |  | 3 |
| Aversive-Racism Theory |  |  |  | 1 |  |  |  |  |  |  |  | 1 |
| Baddeley's Working Memory Model |  |  |  | 1 |  |  |  |  |  |  |  | 1 |
| Balance Theory (Balance Model Of Self-Regulation) |  |  |  |  | 2 |  |  |  |  |  | 1 | 3 |
| Bayesian Decision Theory |  | 2 |  |  | 1 |  |  |  |  |  |  | 3 |
| Behavioral Immune System |  |  |  |  |  |  |  |  | 1 | 1 |  | 2 |
| Behaviorism Theory (Skinner) |  |  |  |  |  |  |  |  | 1 |  |  | 1 |
| Belongingness Self-Regulation Theory | 1 |  |  |  |  |  |  |  |  |  |  | 1 |
| Bilingual Advantage Theory |  |  |  |  |  |  | 1 |  |  |  |  | 1 |
| Binocular Theory Of Stereopsis |  |  |  |  | 1 |  |  |  |  |  |  | 1 |
| Bioinformational Theory Of Emotional Imagery |  |  |  | 1 |  |  |  |  |  |  |  | 1 |
| Biological Markets Theory |  |  |  |  |  |  |  |  |  | 1 |  | 1 |
| Biological Sensitivity To Context |  |  |  | 1 | 1 | 1 |  |  |  |  |  | 3 |
| Biological Theory Of The Big 5 |  |  |  |  |  |  |  |  |  |  |  | 0 |
| Biopsychosocial Model (of Challenge And Threat) |  |  |  |  |  |  |  | 1 |  |  |  | 1 |
| Bis/Bas | 1 |  | 1 |  | 1 |  |  |  |  |  |  | 3 |
| Boolean Map Theory |  | 1 |  | 1 |  |  | 1 |  |  |  |  | 3 |
| Broaden-And-Build Theory Of Positive Emotions | 1 |  |  |  | 1 |  |  |  |  |  |  | 2 |
| Broken Windows Theory |  |  |  |  | 1 |  |  | 1 |  | 1 |  | 3 |
| Brunswikian Lens Model Of Individual Differences |  |  |  |  |  |  |  | 1 |  |  |  | 1 |
| Catharsis Theory |  | 1 |  |  |  |  |  |  |  |  |  | 1 |
| Challenge-And-Threat Theory | 1 |  |  |  |  |  |  |  |  |  |  | 1 |
| Climato-Economic Theory Of Cultural Change |  |  |  |  |  |  |  |  | 1 |  |  | 1 |
| Cognitive Ability Theory |  |  | 1 |  |  |  |  |  |  |  |  | 1 |
| Cognitive Development Theory |  |  |  |  |  |  |  |  |  | 1 |  | 1 |
| Cognitive Dissonance |  | 3 | 1 | 1 | 1 |  | 2 |  |  |  |  | 8 |
| Cognitive Film Theory |  | 1 |  |  |  |  |  |  |  |  |  | 1 |
| Cognitive Hierarchy Theory |  |  |  | 1 |  |  |  |  |  |  |  | 1 |
| Cognitive Neoassociation Theory |  |  |  |  | 1 | 1 |  |  |  |  |  | 2 |
| Collective Effervescence (Durkheim) |  |  |  |  |  |  |  |  |  |  | 1 | 1 |
| Common Coding Theory |  | 1 |  |  |  |  |  |  |  |  |  | 1 |
| Communication Theory (Shannon's) |  |  |  |  | 1 |  |  |  | 1 |  |  | 2 |
| Competition Between Verbal And Implicit Systems (COVIS) |  | 2 |  |  |  |  |  |  |  |  |  | 2 |
| Concealed Information Test Theory |  |  |  |  |  |  |  |  |  |  | 1 | 1 |
| Conceptual-Metaphor Theory |  |  | 2 |  |  |  |  |  |  |  |  | 2 |
| Concreteness Fading Theory |  |  |  |  |  | 1 |  |  |  |  |  | 1 |
| Conflict Monitoring Theory |  |  |  |  |  | 1 |  |  |  |  |  | 1 |
| Conflict Theory |  |  |  |  |  | 1 |  |  |  |  |  | 1 |
| Conflict Theory Of The Evolution Of Genomic Imprinting |  |  |  |  |  |  |  |  | 1 |  |  | 1 |
| Confluence Theory (Model) |  |  |  |  |  |  |  |  |  |  | 1 | 1 |
| Constrict Theory |  |  |  |  |  | 1 |  |  |  |  |  | 1 |
| Construal-Level Theory (Psychological Distance) | 2 |  |  | 4 | 2 | 2 | 3 |  | 1 |  |  | 14 |
| Contact Theory (Intergroup) |  |  |  | 1 |  | 1 |  |  |  |  |  | 2 |
| Contagion Theory | 1 |  |  |  |  |  |  |  |  |  |  | 1 |
| Contingent Automaticity (Conditional Automaticity) |  |  |  |  |  | 1 |  |  |  |  |  | 1 |
| Control Theory |  |  |  |  |  |  |  | 1 |  |  |  | 1 |
| Costly-Signaling Theory |  |  |  |  |  |  |  |  | 1 |  |  | 1 |
| Counterfactual Simulation Model |  |  |  |  |  |  |  |  | 1 |  |  | 1 |
| Cue Integration Theory |  |  |  |  |  |  | 1 |  |  |  |  | 1 |
| Culture As Situated Cognition |  |  |  |  | 1 |  |  |  |  |  |  | 1 |
| Decision Field Theory |  |  |  |  |  |  | 1 |  | 1 | 1 |  | 3 |
| Decision-Affect Theory |  |  | 1 |  |  |  |  |  |  |  |  | 1 |
| Delay Discounting |  |  |  |  |  |  | 1 |  |  |  |  | 1 |
| Desensitization Theory |  |  |  |  |  | 1 |  |  |  |  |  | 1 |
| Dialogic Theory |  |  |  |  |  |  |  |  | 1 |  |  | 1 |
| Differential Susceptibility |  |  |  |  | 1 | 1 | 1 | 1 |  |  |  | 4 |
| Digital Goldilocks Hypothesis |  |  |  |  |  |  |  |  | 1 |  |  | 1 |
| Disjunctive Motivation Model |  | 1 |  |  |  |  |  |  |  |  |  | 1 |
| Dissociable-Subsystems Theory | 1 |  |  |  |  |  |  |  |  |  |  | 1 |
| Divisive Normalization |  |  |  |  |  |  |  | 1 |  |  |  | 1 |
| Double Filtering By Frequency |  | 1 |  |  |  |  |  |  |  |  |  | 1 |
| Drift-Diffusion Model |  |  |  |  |  |  | 1 |  |  |  |  | 1 |
| Dual-Process Theory (Reasoning) | 1 |  |  | 1 |  |  |  | 1 | 1 |  |  | 4 |
| Dual-Process Theory Of Moral Judgement |  |  |  | 1 |  |  |  |  |  |  |  | 1 |
| Duplex Vision Theory |  | 1 |  |  |  |  |  |  |  |  |  | 1 |
| Dyadic Meta-Accuracy |  |  |  |  |  |  |  |  | 1 |  |  | 1 |
| Dynamic Field Theory | 1 |  |  |  |  |  |  |  |  |  |  | 1 |
| Dynamic Touch Theory |  |  |  |  | 1 |  |  |  |  |  |  | 1 |
| Effortful Adjustment |  |  |  |  |  |  |  |  |  |  | 1 | 1 |
| Ego-Depletion |  | 1 | 1 |  | 1 | 1 |  | 1 |  |  |  | 5 |
| Embodied Social Communication Theory |  |  |  | 1 |  |  |  |  |  |  |  | 1 |
| Emotion As Social Information |  | 1 |  |  |  |  |  |  |  |  |  | 1 |
| Emotion Feedback Theory |  |  |  |  |  | 1 |  |  |  |  |  | 1 |
| Emotional Processing Theory |  |  |  | 1 |  |  |  |  |  |  |  | 1 |
| Empirical Ranking Theory |  |  |  |  |  |  |  |  | 1 |  |  | 1 |
| Equity Theory | 1 |  |  | 1 |  |  |  |  |  |  |  | 2 |
| Error Management Theory |  |  |  | 1 | 1 |  | 2 |  | 1 |  |  | 5 |
| Evaluability Theory |  |  |  |  | 1 |  | 1 |  | 1 |  |  | 3 |
| Event Segmentation Theory |  |  | 1 |  | 1 |  |  |  |  |  |  | 2 |
| Evolution Theory |  | 2 | 1 |  |  | 2 | 1 |  |  |  |  | 6 |
| Evolutionary Theory Of PMS | 1 |  |  |  |  |  |  |  |  |  |  | 1 |
| Evolutionary Theory Of Socialization |  |  |  |  |  |  |  | 1 |  |  |  | 1 |
| Expectancy Theory (Vroom) |  |  |  |  |  |  |  |  | 1 |  |  | 1 |
| Expectancy X Value Theory |  |  | 1 | 1 |  |  |  |  | 1 | 1 |  | 4 |
| Expected Utility Theory | 1 |  |  | 2 |  | 1 |  |  | 2 |  |  | 6 |
| Extended-Now Theory |  |  |  | 1 |  |  |  |  |  |  |  | 1 |
| Facultative Calibration Theory |  |  |  |  |  |  | 1 |  |  |  |  | 1 |
| False-Signaling Theory Of Hypocrisy |  |  |  |  |  |  |  |  | 1 |  |  | 1 |
| Family Niche Theory |  |  |  |  |  |  |  |  | 1 |  |  | 1 |
| Fearlessness Theory |  |  |  |  | 1 |  |  |  |  |  |  | 1 |
| Feature Integration Theory |  | 4 |  |  |  |  |  | 1 |  |  |  | 5 |
| Five-Factor Model (Of Traits, Of Personality) |  |  |  | 1 | 1 | 2 |  |  |  |  |  | 4 |
| Fluid-Crystallized Theory (Of Intelligence) |  |  |  |  | 1 |  |  |  |  | 1 |  | 2 |
| Focus Theory Of Norms |  |  |  |  |  |  |  |  | 1 |  |  | 1 |
| Frustration-Aggression Theory |  |  |  |  | 1 |  |  |  |  |  |  | 1 |
| Fuzzy-Trace Theory | 1 |  | 2 | 1 | 1 | 1 |  |  |  |  |  | 6 |
| G Theory (Of Intelligence) |  |  |  |  | 1 |  |  |  |  | 1 | 1 | 3 |
| Game Theory |  |  |  | 1 | 1 | 5 | 1 | 2 | 1 |  |  | 11 |
| Gaussian Random-Field Theory |  |  |  |  | 1 |  |  |  |  | 1 |  | 2 |
| Gene-Culture Coevolution Theory |  |  |  |  |  |  |  |  | 1 |  |  | 1 |
| Generate-Recognize Theory | 1 |  |  |  |  |  |  |  |  |  |  | 1 |
| Genetic Maturation Hypothesis |  |  |  |  | 1 |  |  |  |  |  |  | 1 |
| Genetic Set Point Hypothesis |  |  |  |  | 1 |  |  |  |  |  |  | 1 |
| Genotype Environment Transaction Hypothesis |  |  |  |  | 1 |  |  |  |  |  |  | 1 |
| Global (Neuronal) Workspace Theory (Of Consciousness) |  | 1 | 1 |  |  | 1 |  |  |  | 1 |  | 4 |
| Goal Congruity Theory |  |  |  |  |  |  |  |  | 1 | 1 |  | 2 |
| Goal Gradient Theory |  |  |  |  |  |  | 1 |  |  |  |  | 1 |
| Graph Theory | 1 |  |  |  |  |  |  |  |  |  |  | 1 |
| Gray's Theory | 1 |  |  |  |  |  |  |  |  |  |  | 1 |
| Hard Interface Theory |  |  | 1 |  |  |  |  |  |  |  |  | 1 |
| Hierarchical Integration Theory |  | 1 |  |  |  |  |  |  |  |  |  | 1 |
| Hormone Diversity Fit |  |  |  |  |  |  |  |  |  | 1 |  | 1 |
| I3 Theory |  |  |  |  |  |  |  |  |  |  | 1 | 1 |
| Identity-Based Motivation Theory |  |  |  |  |  |  | 1 |  |  |  |  | 1 |
| Ideomotor Theory |  | 2 |  |  |  | 1 |  |  |  |  |  | 3 |
| Implicit Theory (Mindset Theories; Of Personality Or Intelligence) |  | 1 | 2 | 1 |  |  |  | 1 |  | 3 |  | 8 |
| Inattentional Amnesia Theory |  |  |  |  |  | 1 |  |  |  |  |  | 1 |
| Inclusive Fitness Theory |  | 1 |  |  |  |  |  |  |  |  |  | 1 |
| Inferiority Complex Theory |  |  |  |  |  |  |  |  |  | 1 |  | 1 |
| Information Integration Theory (Consciousness) |  |  |  |  |  |  |  |  |  | 1 |  | 1 |
| Information Theory | 1 | 1 |  |  | 1 |  |  |  | 1 |  |  | 4 |
| Information-Gap Theory | 1 |  |  |  |  |  |  |  |  |  |  | 1 |
| Inhibitory Theory (Inhibition Theory Of Aging) |  |  | 1 |  |  | 1 |  |  |  |  |  | 2 |
| Integrated Theory Of Numerical Development |  |  |  | 1 |  |  |  |  |  |  |  | 1 |
| Interdependence Theory |  |  | 2 |  | 2 |  |  | 1 |  |  |  | 5 |
| Interference Theory |  | 1 |  |  |  |  |  |  |  |  |  | 1 |
| Intergroup Image Theory | 1 |  |  |  |  |  |  |  |  |  |  | 1 |
| Interpersonal Circumplex Theory |  |  |  |  | 1 |  |  |  |  |  |  | 1 |
| Investment Theory |  |  |  |  |  |  |  |  |  |  | 1 | 1 |
| Ironic Process Theory |  | 1 |  |  |  |  |  |  |  |  |  | 1 |
| Item Response Theory |  |  | 2 | 1 |  | 4 | 1 |  | 1 | 1 | 1 | 11 |
| Jamesian (Bodily-Feedback) Theory |  | 1 |  |  |  |  |  |  |  |  |  | 1 |
| John Henryism Theory |  |  |  |  | 1 |  |  |  |  |  |  | 1 |
| Just-World Theory |  | 1 | 1 | 1 |  |  |  |  |  |  |  | 3 |
| Kinesthetic Imagery |  |  |  |  | 1 |  |  |  |  |  |  | 1 |
| Kosslyn's Theory | 1 |  |  |  |  |  |  |  |  |  |  | 1 |
| Lavie's Load Theory | 1 |  |  |  |  |  |  |  |  |  |  | 1 |
| Law Of Prior Entry |  |  |  |  |  |  |  |  | 1 |  |  | 1 |
| Leaky Competing Accumulators Model |  |  |  |  | 1 |  | 1 |  |  | 1 |  | 3 |
| Learning Theory |  |  |  |  | 1 |  |  |  |  |  |  | 1 |
| Lewinian Goal Theory | 1 |  |  |  |  |  |  |  |  |  |  | 1 |
| Life History Theory |  | 2 |  |  | 3 | 2 | 1 | 4 |  | 2 | 1 | 15 |
| Life-Span Developmental Theory |  |  |  |  |  |  |  | 1 |  |  |  | 1 |
| Livability |  |  |  |  |  |  |  |  |  | 1 |  | 1 |
| Maslow's Theory Of The Hierarchy Of Needs |  |  |  |  |  |  |  |  | 1 |  |  | 1 |
| Mathematical Cognition (Lewis, Matthews & Hubbard) |  |  |  |  |  |  |  | 1 |  |  |  | 1 |
| Mental Number Line Theory |  |  | 1 |  |  |  |  |  |  |  |  | 1 |
| Mind-Set Theory (Gollwitzer's) |  |  |  | 1 |  |  |  |  |  |  |  | 1 |
| Misattribution Of Arousal |  |  |  |  | 1 |  |  |  |  |  |  | 1 |
| Mood Management Theory |  |  |  |  | 1 |  |  |  |  |  |  | 1 |
| Mood Repair Motives (Clark & Isen's) |  |  |  |  | 1 |  |  |  |  |  |  | 1 |
| Moral Deservingness Theory |  |  |  |  |  |  |  |  | 1 |  |  | 1 |
| Moral Foundations |  |  |  |  | 1 | 1 |  | 1 | 1 | 1 | 1 | 6 |
| Moral Self-Licencing |  |  |  |  | 1 |  |  |  |  |  |  | 1 |
| Motivated Social Cognition Theory |  |  |  |  |  |  |  | 1 |  |  |  | 1 |
| Motivational Intensity Theory |  |  |  |  |  |  |  |  | 1 |  |  | 1 |
| Motor Theory Of Speech Perception |  | 1 |  |  |  | 1 |  | 1 |  |  |  | 3 |
| Multialternative Decision Field Theory |  |  |  |  | 1 |  |  |  |  |  |  | 1 |
| Multiattribute Linear Ballistic Accumulator Model |  |  |  |  |  |  |  |  | 1 |  |  | 1 |
| Multiple Systems Theory |  |  |  |  |  | 1 |  |  |  |  |  | 1 |
| Multiprocess Theory |  |  |  |  | 1 |  |  |  |  |  |  | 1 |
| Multiscale Context Model |  |  |  |  |  | 1 |  |  |  |  |  | 1 |
| Natural Pedagogy Theory |  |  |  |  | 1 |  |  |  |  |  |  | 1 |
| Need Fulfilment Theory |  |  |  |  |  |  |  |  |  | 1 |  | 1 |
| Network Reset Theory |  |  |  |  |  |  |  |  |  |  | 1 | 1 |
| Neural-Synchrony Theory |  |  |  |  | 1 |  |  |  |  |  |  | 1 |
| Neuropsychological Theory Of Anxiety | 1 |  |  |  |  |  |  |  |  |  |  | 1 |
| New Look |  | 1 |  |  |  |  |  |  |  |  |  | 1 |
| New Theory Of Disuse (Memory) |  |  |  |  |  |  |  | 1 |  |  |  | 1 |
| Norm Theory |  |  | 1 |  |  |  |  |  |  | 1 |  | 2 |
| Norm-Focus Theory | 1 |  |  |  |  |  |  |  |  |  |  | 1 |
| Normative Decision Theory |  |  |  |  |  |  |  |  |  | 1 |  | 1 |
| Object-File Theory |  |  |  |  | 1 |  |  | 1 |  |  |  | 2 |
| Object-Updating Theory | 1 |  |  |  |  |  |  |  |  |  |  | 1 |
| Objectification Theory |  | 1 |  | 1 | 1 |  |  |  |  |  |  | 3 |
| Opponent Process Theory (Solomon) |  |  |  |  | 1 |  |  |  |  |  |  | 1 |
| Opponent Process Theory (Of Color Vision) |  |  |  | 1 | 1 |  |  |  |  |  |  | 2 |
| Optimal Control Theory |  |  |  |  |  |  |  |  | 1 |  |  | 1 |
| Optimal Distinctiveness Theory | 1 |  |  |  |  |  |  |  |  |  |  | 1 |
| Optimal Foraging Theory |  |  |  | 1 |  |  |  |  |  |  |  | 1 |
| Orienting Response Theory |  |  |  |  |  |  |  |  |  |  | 1 | 1 |
| Overjustification Theory |  |  | 1 |  |  |  |  |  |  |  |  | 1 |
| Overlapping Waves Theory |  | 1 |  |  |  |  |  |  |  |  |  | 1 |
| Overwhelming Risk Hypothesis |  |  |  |  |  |  |  |  |  |  | 1 | 1 |
| Ovulatory Shift Hypothesis |  |  |  |  |  | 1 |  |  |  |  |  | 1 |
| Pacherie's Dynamic Framework Of Intention |  |  |  | 1 |  |  |  |  |  |  |  | 1 |
| Parental Investment Theory |  |  | 2 | 1 |  |  |  |  |  |  |  | 3 |
| Parieto-Frontal Integration Theory |  |  | 1 |  |  |  |  |  |  |  |  | 1 |
| Partisan Motivated Reasoning |  |  |  |  |  |  |  |  |  | 1 |  | 1 |
| Pathogen Prevalence Theory |  |  |  |  |  |  | 1 |  |  |  |  | 1 |
| Pavlovian Conditioning (Learning) Theory |  |  |  |  |  |  |  | 1 | 1 |  |  | 2 |
| Perception Action Dissociation |  |  | 1 |  |  |  |  |  |  |  |  | 1 |
| Perceptual Load Theory |  |  | 1 | 1 |  |  |  |  |  | 1 |  | 3 |
| Perceptual Set Theory |  |  |  |  | 1 |  |  |  |  |  |  | 1 |
| Perceptual-Symbol-Systems Theory |  |  | 1 |  |  |  |  |  |  |  |  | 1 |
| PERSON Model |  |  |  | 1 |  |  |  |  |  |  |  | 1 |
| Piaget’s (theory) |  |  |  |  | 1 |  |  |  |  |  |  | 1 |
| Polyvagal Theory |  |  | 1 |  | 1 |  | 1 | 2 |  |  |  | 5 |
| Positive Illusion Theory | 1 |  |  |  |  |  |  |  |  |  |  | 1 |
| Possible-Selves Theory | 1 |  |  |  |  |  |  |  |  |  |  | 1 |
| Predictive Control Theory |  |  |  |  |  |  |  |  | 1 |  |  | 1 |
| Premotor Theory Of Attention | 1 |  | 1 |  |  |  |  |  |  |  |  | 2 |
| Prospect Theory (Also Cumulative Prospect) | 3 |  | 2 | 2 |  | 4 | 2 | 2 | 2 | 1 | 3 | 21 |
| Protection-Motivation Theory |  |  |  |  |  |  |  | 1 |  |  |  | 1 |
| Prototype-Based Theory Of Face Representation | 1 |  |  |  |  |  |  |  |  |  |  | 1 |
| Psychological Fit (Person-Environment Fit) |  |  |  |  |  |  |  | 1 |  | 1 |  | 2 |
| Psychological Refractory Period |  |  |  |  | 1 |  |  |  |  |  |  | 1 |
| Quantal-Response Equilibrium |  |  |  | 1 |  |  |  |  |  |  |  | 1 |
| Query Theory |  | 1 |  |  | 1 |  |  |  |  |  |  | 2 |
| Queueing Theory |  |  |  | 1 |  |  |  |  |  |  |  | 1 |
| Range-Frequency Theory | 1 | 1 |  |  |  | 1 |  | 1 |  |  |  | 4 |
| Ratio Of Strengths Model (Luce) |  |  |  |  | 1 |  |  |  |  |  |  | 1 |
| Rational Choice Theory |  |  |  | 1 | 1 |  |  |  |  |  |  | 2 |
| Rawl's Theory Of Justice |  |  |  | 1 |  |  |  |  |  |  |  | 1 |
| Reactance Theory (Psychological Reactance) |  |  | 1 | 1 |  |  |  |  |  |  |  | 2 |
| Realistic Group Conflict Theory |  |  |  |  |  |  |  |  |  | 1 |  | 1 |
| Reappropriation (Theoretical Model Of) |  |  |  |  | 1 |  |  |  |  |  |  | 1 |
| Reconsolidation Theory |  |  |  |  | 1 |  | 1 |  |  |  |  | 2 |
| Reentrant Theory Of OSM | 1 |  |  |  |  |  |  |  |  |  |  | 1 |
| Reference Dependent Theory Of Riskless Choice |  |  |  |  | 1 |  |  |  |  |  |  | 1 |
| Regret Theory |  |  | 1 |  |  |  |  |  |  |  |  | 1 |
| Regulatory Focus Theory |  |  |  |  | 1 | 1 |  |  |  |  | 1 | 3 |
| Reinforcement Learning |  | 2 |  |  |  |  |  | 1 |  |  |  | 3 |
| Relational Complexity Theory |  |  |  |  | 1 |  |  |  |  |  |  | 1 |
| Relational Models Theory |  | 1 |  |  |  |  |  |  |  |  |  | 1 |
| Relational Primacy Theory |  |  |  |  | 1 |  |  |  |  |  |  | 1 |
| Relational Shift Theory |  |  |  |  | 1 |  |  |  |  |  |  | 1 |
| Representation Theory Of Forgetting |  |  |  |  |  |  |  | 1 |  |  |  | 1 |
| Rescorla-Wagner Theory |  | 1 |  |  |  |  |  |  |  |  |  | 1 |
| Resource Conservation Theory |  |  | 1 |  |  |  |  |  |  |  |  | 1 |
| Resource Depletion (And Recovery) Theory |  |  |  |  | 1 |  |  |  |  |  | 1 | 2 |
| Resource Dilution Model |  |  |  |  |  |  |  |  |  |  | 1 | 1 |
| Resource Substitution Theory |  |  |  |  |  |  |  |  |  |  | 1 | 1 |
| Response Modulation Theory |  |  | 1 |  |  |  |  |  |  |  |  | 1 |
| Responsiveness Theory |  |  |  |  |  |  |  | 1 |  |  |  | 1 |
| Retrieving Effectively From Memory Theory |  |  |  | 1 |  |  |  |  |  |  |  | 1 |
| Risk Regulation Theory |  |  |  |  |  |  | 1 |  |  |  |  | 1 |
| Risk-Sensitive Foraging Theory |  | 1 |  |  |  |  |  |  |  |  |  | 1 |
| Role Congruity Theory | 1 |  |  |  |  |  |  |  |  |  |  | 1 |
| Scalar Expectancy Theory |  |  |  |  |  |  | 1 |  |  |  |  | 1 |
| Scale Distortion (Anchoring) |  |  |  |  |  |  |  |  |  |  | 1 | 1 |
| Scale Granularity (Anchoring) |  |  |  |  |  |  |  | 1 |  |  |  | 1 |
| Schema Discrepancy Theory |  |  | 1 |  |  |  |  |  |  |  |  | 1 |
| Schema Theory (Schmidt) |  |  |  |  |  |  |  |  |  |  | 1 | 1 |
| Script Theory |  |  |  |  |  | 1 |  |  |  |  |  | 1 |
| Secondary-Anti-Semitism-Theory | 1 |  |  |  |  |  |  |  |  |  |  | 1 |
| Self-Affirmation Theory | 2 |  | 2 | 2 | 1 | 1 |  | 1 |  | 1 |  | 10 |
| Self-Attention Network |  |  |  |  |  |  |  |  | 1 |  |  | 1 |
| Self-Categorization Theory |  |  |  |  |  |  |  |  |  |  | 1 | 1 |
| Self-Completion Theory | 1 |  | 1 |  |  |  |  |  |  |  |  | 2 |
| Self-Congruity Theory |  |  |  |  |  |  |  |  |  |  | 1 | 1 |
| Self-Construal |  |  |  |  | 1 |  |  |  |  |  |  | 1 |
| Self-Determination Theory (Or Its Mini-Theories) |  | 1 | 2 | 3 | 1 |  |  |  | 1 |  |  | 8 |
| Self-Discrepancy Theory |  |  |  |  |  |  |  |  |  | 1 |  | 1 |
| Self-Efficacy Theory |  | 1 |  |  |  |  |  |  |  |  |  | 1 |
| Self-Perception Theory |  | 1 |  | 2 | 2 | 1 |  | 1 |  |  |  | 7 |
| Self-Regulation Theory |  | 1 |  |  |  |  |  |  | 1 |  |  | 2 |
| Self-Validation Theory Of Judgement |  |  |  |  |  |  |  | 1 |  |  |  | 1 |
| Self-Verification Theory | 1 | 2 |  |  |  | 1 |  |  |  |  |  | 4 |
| Sensation Seeking (Stimulation Seeking) |  |  |  |  | 1 |  |  |  |  |  |  | 1 |
| Sensory Recruitment Hypothesis |  |  |  |  |  |  |  |  |  | 1 |  | 1 |
| Set Point Theory Of Wellbeing |  |  |  |  |  |  |  | 1 |  |  |  | 1 |
| Set Theory |  |  |  |  |  |  |  |  |  | 1 |  | 1 |
| Sexual Economics Theory |  |  |  |  |  | 2 |  |  |  |  |  | 2 |
| Sexual Selection Theory |  |  |  |  |  |  | 2 |  | 1 | 1 |  | 4 |
| Shared Reality Theory |  |  |  |  |  |  |  |  |  | 1 |  | 1 |
| Signal Detection Theory | 2 | 2 | 2 | 3 | 2 | 6 | 1 | 2 | 3 | 2 | 2 | 27 |
| Similarity Attraction Theory |  |  |  |  |  | 1 |  |  |  |  |  | 1 |
| Simulation Theory | 1 |  | 1 |  |  |  |  |  |  |  |  | 2 |
| Situated-Focus Theory Of Power |  |  |  | 1 |  |  |  | 1 |  |  |  | 2 |
| Sleep-Related Memory Improvement |  | 1 |  |  |  |  |  |  |  |  |  | 1 |
| Social Change And Human Development (Greenfield) |  |  |  |  | 1 |  | 1 |  |  |  |  | 2 |
| Social Cognitive Theory |  |  |  |  |  | 1 |  |  | 1 |  |  | 2 |
| Social Comparison Theory |  | 1 |  |  |  |  |  |  |  |  |  | 1 |
| Social Domain Theory |  |  |  |  |  |  | 1 |  |  |  |  | 1 |
| Social Dominance Theory | 1 |  |  | 2 | 1 | 1 | 1 |  |  |  | 1 | 7 |
| Social Exchange Theory |  |  |  |  |  |  |  |  |  |  | 1 | 1 |
| Social Functional Approach (Personality & Intentions) |  |  |  |  |  |  |  | 1 |  |  |  | 1 |
| Social Heuristics Hypothesis |  |  |  |  |  |  |  | 1 |  |  |  | 1 |
| Social Information Processing Theory |  |  |  |  |  | 1 |  |  |  |  |  | 1 |
| Social Justice Model |  |  | 1 |  |  |  |  |  |  |  |  | 1 |
| Social Learning Theory |  |  |  |  |  | 2 | 1 |  | 1 | 1 |  | 5 |
| Social Monitoring Theory | 1 |  |  |  |  |  |  |  |  |  |  | 1 |
| Social Role Theory |  |  |  | 1 |  |  | 1 |  | 1 | 1 |  | 4 |
| Social Structure Theory (Social Structural Theory) |  |  |  | 1 |  |  | 1 |  |  |  |  | 2 |
| Social-Identity Theory | 2 | 2 | 2 | 2 | 1 | 1 |  |  |  | 3 |  | 13 |
| Social-Impact Theory | 1 |  |  | 1 |  |  |  |  |  |  |  | 2 |
| Social-Investment Theory |  |  |  |  | 1 | 2 |  |  |  |  |  | 3 |
| Social-Norms Theory | 1 |  |  |  |  |  |  |  |  |  |  | 1 |
| Socioemotional Selectivity Theory | 1 |  |  | 1 |  |  |  |  |  |  |  | 2 |
| Spatial-Envelope Theory | 1 |  |  |  |  |  |  |  |  |  |  | 1 |
| Standard Operating Procedures Theory |  |  |  |  |  |  |  |  |  | 1 |  | 1 |
| Status Inconsistency Theory |  |  |  |  | 1 |  |  |  |  |  |  | 1 |
| Stereotype Embodiment Theory |  |  |  |  |  | 1 |  |  |  |  |  | 1 |
| Stereotype Threat |  |  |  |  |  |  |  |  |  | 1 |  | 1 |
| Stress Appraisal Theory |  |  |  |  |  |  |  | 1 |  |  |  | 1 |
| Stress Generation Model |  |  | 1 |  |  |  |  |  |  |  |  | 1 |
| Support Theory |  |  |  | 1 |  |  |  |  |  | 1 |  | 2 |
| Surprisal Theory |  |  | 1 |  |  |  |  |  |  |  |  | 1 |
| Symbolic Racism Theory |  |  |  |  | 1 |  |  |  |  |  |  | 1 |
| Syntactic Bootstrapping Theory | 1 |  |  |  |  |  |  |  |  |  |  | 1 |
| Syntactic Theory |  | 1 |  |  |  |  |  |  |  |  |  | 1 |
| System Justification Theory | 1 | 2 | 1 | 1 | 3 | 1 | 2 |  |  |  | 1 | 12 |
| Teleological Stance Theory | 1 |  |  |  |  | 1 |  |  |  |  |  | 2 |
| Temporal Motivation Theory |  |  |  |  |  | 1 |  |  |  |  |  | 1 |
| Tend And Befriend Model |  |  |  | 1 | 1 |  | 2 |  |  |  |  |  |
| Tension Reduction Hypothesis | 1 |  |  |  |  |  |  |  |  |  |  | 1 |
| Terror Management Theory | 1 |  | 2 | 1 | 1 | 3 | 1 | 1 |  |  |  | 10 |
| Test-Interference Theory |  | 1 |  |  |  |  |  |  |  |  |  | 1 |
| Theory Of Emerging Adulthood |  |  |  |  | 1 |  |  |  |  |  |  | 1 |
| Theory Of Event Coding | 1 | 1 | 1 |  | 1 |  | 1 |  |  | 1 |  | 6 |
| Theory Of Group Productivity (Steiner's) |  |  |  |  | 1 |  |  |  |  |  |  | 1 |
| Theory Of Inequality |  |  |  |  |  |  |  |  |  | 1 |  | 1 |
| Theory Of List Memory |  |  |  | 1 |  |  |  |  |  |  |  | 1 |
| Theory Of Magnitude | 1 |  | 1 |  |  |  |  |  |  |  |  | 2 |
| Theory Of Personal Identity |  |  |  |  |  |  |  | 1 |  |  |  | 1 |
| Theory Of Planned Behavior |  | 1 |  |  |  |  |  | 1 |  |  |  | 2 |
| *Theory Of Relativity* (not a psychological theory) |  | 1 |  |  |  |  |  |  |  |  |  | 1 |
| Theory Of Socialization |  | 1 |  |  |  |  |  |  |  |  |  | 1 |
| Theory Of Survival Processing |  |  |  |  |  | 1 |  |  |  |  |  | 1 |
| Time On Task Theory Of Learning |  |  |  |  |  | 1 |  |  |  |  |  | 1 |
| Transition Theory |  |  |  | 1 |  |  |  |  |  |  |  | 1 |
| Truth Default Theory |  |  |  |  |  | 1 |  |  |  |  |  | 1 |
| Two Visual Streams Theory |  |  |  |  | 1 |  |  |  |  |  |  | 1 |
| Two-Factor Theory |  | 1 |  |  |  |  |  | 1 |  |  |  | 2 |
| Uncanny Valley |  | 1 |  |  |  |  |  |  |  |  |  | 1 |
| Uncertainty Management |  |  |  |  | 1 |  |  |  |  |  |  | 1 |
| Unconscious Thought Theory | 2 | 1 | 2 |  | 1 |  |  |  |  |  |  | 6 |
| Unique Attribute Hypothesis |  | 1 |  |  |  |  |  |  |  |  |  | 1 |
| Values Theory |  |  |  | 1 |  |  |  |  |  |  |  | 1 |
| Warm Glow Giving |  |  |  |  |  |  |  |  |  | 1 |  | 1 |
| Whole-Trait Theory |  |  |  | 1 |  |  |  |  |  |  |  | 1 |

**S8 Table. Total number of studies using the word hypothesis correctly and incorrectly according to year.**

| Year | Total  Articles | Articles Using  Hypothesis | Articles Using  Hypothesis Incorrectly |
| --- | --- | --- | --- |
| 2009 | 192 | 106 | 33 |
| 2010 | 284 | 175 | 31 |
| 2011 | 232 | 151 | 20 |
| 2012 | 213 | 120 | 5 |
| 2013 | 304 | 188 | 26 |
| 2014 | 242 | 139 | 18 |
| 2015 | 178 | 116 | 20 |
| 2016 | 144 | 88 | 18 |
| 2017 | 145 | 99 | 6 |
| 2018 | 156 | 109 | 10 |
| 2019 | 135 | 95 | 15 |
